# Next-Generation Sequencing Analysis of Cellular Response to Influenza B Virus Infection

**DOI:** 10.3390/v12040383

**Published:** 2020-03-31

**Authors:** Zizhang Sheng, Chen Huang, Runxia Liu, Yicheng Guo, Zhiguang Ran, Feng Li, Dan Wang

**Affiliations:** 1 Zukerman Institute of Mind Brain Behavior, Columbia University, New York, NY 10027, USA; yg2521@cumc.columbia.edu; 2 Department of Biology and Microbiology, South Dakota State University, Brookings, SD 57007, USA; Chen.Huang@sdstate.edu (C.H.); Runxia.Liu@sdstate.edu (R.L.); Feng.Li@sdstate.edu (F.L.)

**Keywords:** Influenza B virus, innate immune response, RNA-Seq, infection

## Abstract

Influenza B virus (IBV) is a respiratory pathogen that infects humans and causes seasonal influenza epidemics. However, cellular response to IBV infection in humans and mechanisms of host-mediated restriction of IBV replication are not thoroughly understood. In this study, we used next-generation sequencing (NGS) to perform transcriptome profiling of IBV-infected human lung epithelial A549 cells at 0, 6, 12, and 24 h post infection (hpi) and characterized the cellular gene expression dynamics. We observed that more than 4000 host genes were differentially regulated during the study period, which included up regulation of genes encoding proteins, having a role in the innate antiviral immune responses, immune activation, cellular metabolism, autophagy, and apoptosis, as well as down regulation of genes involved in mitosis and cell proliferation. Further analysis of RNA-Seq data coupled with RT-qPCR validation collectively showed that double-strand RNA recognition pathways, including retinoic acid-inducible gene I (RIG-I) and Toll-like receptor 3 (TLR3), were substantially activated following IBV infection. Taken together, these results provide important initial insights into the intimate interaction between IBV and lung epithelial cells, which can be further explored towards elucidation of the cellular mechanisms in restriction or elimination of IBV infections in humans.

## 1. Introduction

Influenza viruses are classified as types A, B, and C according to their distinct antigenic properties residing in two major structural proteins (matrix 1 and nucleocapsid) [1]. Recently, a new type of influenza virus with bovine as a primary reservoir, officially designated influenza type D, has been described [2,3]. Among these influenza viruses, only influenza A and B viruses (IAV and IBV) are of medical importance. It has been well established that humans are the primary host and reservoir of IBV [4,5,6,7], although sporadic infection episodes of IBVs have been described in seals and pigs [8,9]. In contrast, IAV circulates in various mammals including humans, swine, and migratory or domestic waterfowl [1]. IBV infection of humans can result in the same spectrum of clinical disease as IAV, ranging from mild to severe respiratory illness requiring immediate hospitalization and medical treatment [7,10]. IBV-associated mortality has been frequently observed in seasonal influenza epidemics particularly in children and adolescents [11,12], and the rate of mortality can be comparable to IAV [13].

As with IAV, IBV contains eight segments of single-stranded negative-sense RNA. IBV evolves at an estimated rate of ~2 × 10^3^ nucleotide substitutions per site per year for hemagglutinin (HA) and neuraminidase (NA) proteins [14,15,16]. This evolutionary rate is approximately 2–3 times slower than those observed in IAVs. Despite the relatively slow evolutionary rate, new antigenic variants of IBV can emerge, largely driven by genetic reassortment of co-circulating IBV strains [6,17,18]. IBV has evolved into two antigenically and genetically distinct lineages, Yamagata and Victoria [19,20]. The antigenic diversity of IBVs has presented a significant challenge for the selection of an appropriate strain for annual vaccine production.

The global burden of annual influenza epidemics involving IBV, coupled with the increasing strong links between IBV and fatal infections in humans [21,22], calls for an urgent need for more basic and clinical investigations of IBV. Despite significant progress on the characterization of host responses to IAV infections in a range of in vitro cell cultures and in vivo animal models, the interplay between IBV infection and host cells especially human epithelial cells remains poorly understood. A high-resolution analysis of dynamic changes in cellular response following IBV infection is also not available. Of many different cellular response pathways, innate immune response is the first line of defense against viral infection. The innate immune response modulates multiple pathways to signaling viral infection, inducing inflammatory reactions, clearing virus, and presenting viral antigens to the host immune system. It also determines how and when the adaptive immune responses will be activated. Thus, a thorough investigation of the innate immune response to IBV infection will shed light on the pathogenicity of IBV and help understand the severity of influenza syndromes and mechanisms of IBV resistance and disease tolerance. Such studies will also further provide clues on how to interfere or prevent IBV infection in humans.

In this study, using next-generation sequencing (NGS) we investigated cellular response to IBV infection in human lung epithelial A549 cells. Significantly, we observed that the double-stranded RNA sensing pathway, consisting of RIG-I and TLR3, played an essential role in detecting IBV infection. Interestingly, in addition to the antiviral innate immune response, we also found that IBV replication resulted in profound changes in gene transcriptions involving other cellular pathways including immune activation, cellular metabolism, autophagy, apoptosis, mitosis, and cell proliferation. Taken together, these results provide important initial insights into the interactions between IBV and human lung epithelial cells, which pave the way for further investigation of cellular mechanism in restriction or elimination of IBV infections in humans.

## 2. Materials and Methods

### 2.1. Virus and Cell Culture

Influenza B/Brisbane/60/2008 virus was used in this study (a gift from Robin Donis at Influenza Division, Center of Disease Control and Prevention, USA). Cell lines acquired from the American Type Culture Collection (ATCC, Manassas, VA, USA) for this work included Human lung alveolar carcinoma epithelial cell line A549 (ATCC^®^ CCL-185) cells, Madin-Darby canine kidney (MDCK) cells (ATCC^®^ CCL-34), and human embryonic kidney cells HEK293T (ATCC^®^ CRL-3216). Cells were maintained in Dulbecco’s minimum essential medium (DMEM) that was supplemented with 10% fetal bovine serum (FBS) (PAA Laboratories Inc., Dartmouth, MA, USA) and penicillin-streptomycin (Life Technologies, Carlsbad, CA, USA) (100 U/mL). MDCK cells were used initially to propagate IBV in T75 tissue culture flasks. Briefly, MDCK cells were cultured to reach only 60% to 70% confluence at the time of infection. After infection, fresh DMEM with 0.5 μg/mL tolylsulfonyl phenylalanyl chloromethyl ketone (TPCK)-treated trypsin (Sigma, Saint Louis, MO, USA) was added for additional 3-day incubation at 37 °C with 5% CO_2_. The infected cell cultures were then frozen and thawed. The supernatant was spun at 500× *g* for 10 min at 4 °C to remove the cellular debris, which was followed by traditional viral titration experiments in MDCK cells according to the method of Reed and Muench [23]. Note that DMEM supplemented with penicillin-streptomycin (Life Technologies, Carlsbad, CA, USA) (200 U/mL) and TPCK-treated trypsin (Sigma, Saint Louis, MO, USA) (1 μg/mL) was used as the virus growth medium.

### 2.2. IBV Infection of A549 Cells and Next-Generation Sequencing

Influenza B/Brisbane/60/2008 virus was used to infect A549 cells at an MOI of 1.0. The viral inoculum was removed after 1 h incubation from cells, which was then followed by PBS washing three times. Fresh DMEM containing penicillin-strepto (200 U/mL) and TPCK-treated trypsin (1 μg/mL) was added into IBV-infected cell for further cultivation. Total cellular RNA was isolated from mock-infected cells (0 hpi) and infected cells at 6, 12, and 24 hpi, respectively, with TRIZOL followed by RNA purification with ethanol. Bioanalyzer 2100 (Agilent, Palo Alto, CA, USA) was used to assess the quality of extracted RNA. RNA that passed the quality test was further processed for cDNA library construction by using a cDNA library prep kit (catalog number FC-122-1001; Illumina Inc.) according to the manufacturer’s instruction. cDNA library construction and all other procedures were conducted in Genomics Core Research Facility (GCRF) at the University of Nebraska-Lincoln (UNL). Briefly, mRNAs were purified from the total RNA using oligo(dT) magnetic beads followed by fragmentation. The resultant mRNAs were reverse transcribed to cDNAs that were subjected to an end repair process followed by ligation to the adapters. After separation in agarose gel through electrophoresis, cDNA fragments with a size of about 200 bp were excised, extracted, and amplified by PCR using two primers that match the ends of adaptors. PCR-enriched samples were then sequenced by an Illumina Genome Analyzer IIx (GA IIx) sequencer in the GCRF at UNL.

Two biological replicates were sequenced for each time point (0, 6, 12, and 24 hpi). The eight samples were barcoded and sequenced in one lane. Each of the eight samples had approximately 5–7 million 100-nucleotide single-end reads. All sequencing results passed the quality control. The raw reads of the eight samples were submitted to NCBI SRA database under Biosample ID: SRS1328599.

### 2.3. Transcriptome Read Processing

The raw read quality was checked using FastQC. The 3’ 5 nucleotides of each read, whose sequencing qualities were low, were removed using fastx_trimmer. GSNAP was then used to map raw reads to human (version: GRCh37.p13) and Influenza B virus genomes [24]. Samtools was used to index bam files and remove PCR duplicates [25]. HTSeq was used to count the number of reads mapped to human genes defined by the Ensemble genome database and viral genes [26].

### 2.4. Differential Gene Expression Analysis

DESeq was used to identify differentially expression human genes [27]. Briefly, samples of 6, 12, and 24 hpi were compared to control samples (0 hpi) to determine whether the expression level of a gene was significantly up- or down-regulated at each time point. To control false discover rate (<10%), we used *P* value adjusted according to the Benjamini–Hochberg method to identify differentially expressed genes (*P* < 0.05). To further remove potential false positives, we removed differentially expressed genes with the changes of expression levels less than ±2 fold.

### 2.5. Gene Ontology and Pathway Enrichment Analysis

We used GSEA database, ClueGO, and STRING database (https://string-db.org/) to identify significantly enriched biological processes, cellular components and molecular functions, and KEGG pathways for the differentially expressed gene sets of each time point [28,29,30] respectively. For the analysis on enrichment of biological processes, a *P* value of 0.05 and normalized false discovery rate of 0.25 were used to identify significantly enriched terms. A P value adjusted according to Bonferroni step down procedure was used to measure the enrichment significance of a GO term (*P* < 0.05) in ClueGo. Parent-child GO terms were fused with Kappa cutoff 0.4. The minimal GO level analyzed was set to 3. The enriched KEGG pathways were identified using a false discover rate of 0.05.

### 2.6. Validation of RNA-Seq Results by RT-qPCR

We performed a standard RT-qPCR to validate the gene expression results observed from next-generation sequencing experiment. Total RNA was extracted from A549 cells as 0, 6, 12, and 24 hpi using TRIzol™ Reagent (Invitrogen, Carlsbad, CA, USA) according to the manufacturer’s instructions, which was then followed by cDNA synthesis with Oligo dT(18) primer (Integrated DNA Technologies, Coralville, IA, USA) and Reverse Transcriptase (Thermo Fisher, Waltham, MA, USA). With Fast SYBR Green Master Mix (Thermo Fisher), qPCR reactions were conducted and read in QuantStudio 6 Flex Real-Time PCR System (Applied Biosystems, Foster City, CA, USA) according to the recommended protocol by the manufacturer. Table S2 summarized sequence information of primers used for expression validation of thirteen genes, including RIG-I, TLR-3, IFN-λ2, IFN-λ3, IFNβ, TNF, ISG15, Mx1, IRF3, IFNα and IFNα-7 (innate immunity pathway); AMBRA1 (autophagy); and CASP10 (apoptosis). Housekeeping gene GAPDH was used as a control in qPCR experiment, while qPCR data were analyzed using the 2-ΔΔCt method [31]. The data were normalized to both the uninfected control and GAPDH and graphed as relative fold change.

## 3. Results

### 3.1. Next-Generation Sequencing of IBV Infected A549 Cells

To investigate the host response to IBV infection, we infected human lung epithelial cells (A549) in duplicate with human IBV strain (Victoria lineage-B/Brisbane/60/2008) at a multiplicity of infection (MOI) of 1.0 [32]. IBV replication was observed at 6 hpi (~1.5 × 10^5^ TCID_50_/mL) and increased over a 24-h period [32]. The principle component analysis showed that the cellular transcriptome profiles of duplicates were highly similar but diverged between time points, suggesting that IBV infection affected gene expression profiles of the host, which had been reported in our previous work [32].

### 3.2. Cellular Processes and Pathways Regulated by IBV Infection

**Figure 1:**
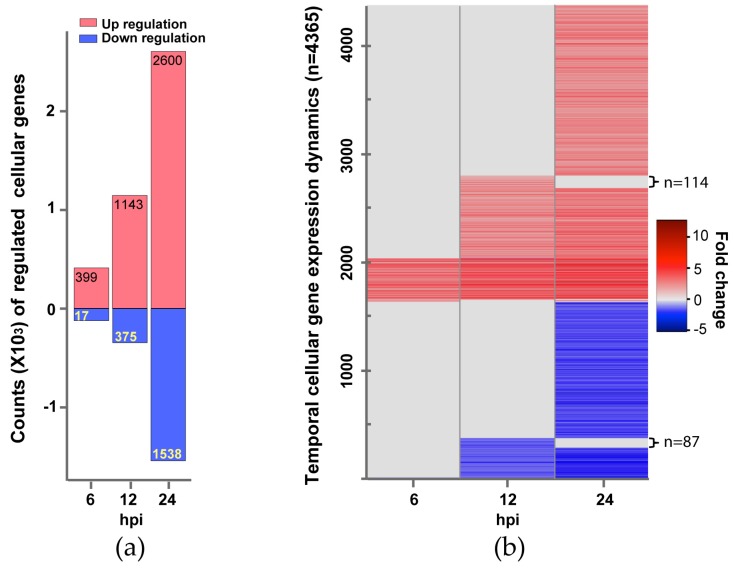
Changes of Influenza B virus (IBV)-regulated cellular genes over time. (**a**) Counts of genes up-regulated (expression level increase is ≥2 fold) or down-regulated (expression level reduction is ≥½) are shown in red or light blue bars, respectively. Differential expression statistical analysis was conducted using DESeq. (**b**) Gene expression Dynamics. A total of 4365 genes showing changed expression at one or more time points (6, 12, and 24 hpi). Each gene’s temporal expression dynamics is displayed as a line with a color according to its expression fold change at a time point post IBV infection. A fold change spectrum is shown as the reference.

To explore cellular responses to IBV infection, we used DESeq program [27] to normalize gene expression levels across time points and to identify human genes significantly regulated by IBV infection (Figure 1). We then estimated enriched biological processes and pathways in up-regulated gene set and down-regulated gene set against the gene ontology (GO) database and KEGG pathway database respectively as a function of time following IBV infection.

**Figure 2:**
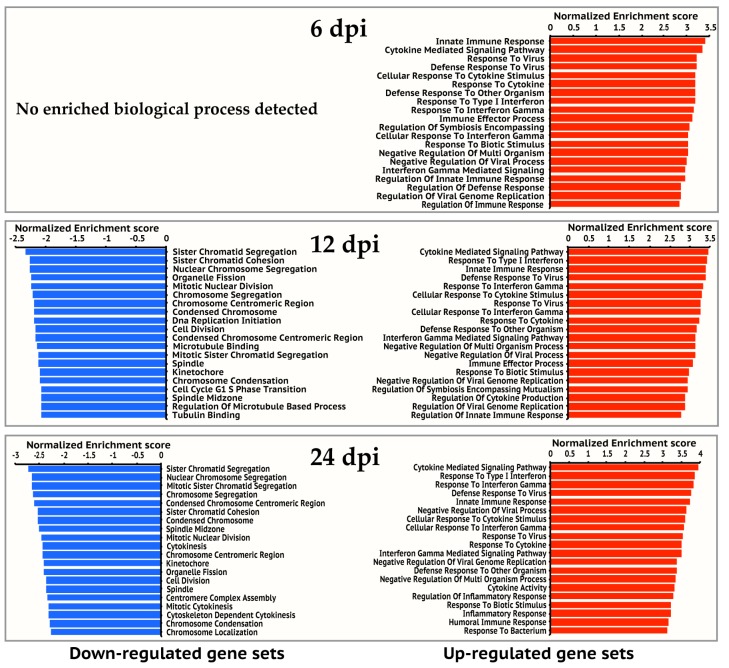
Top 20 enriched gene ontology biological processes for 6, 12, and 24 hpi. Gene ontology enrichment analysis was performed on up-regulated gene set and down-regulated gene set of each time point respectively on the GSEA website. The significantly enriched gene ontology processes were ranked by their enrichment scores and the top 20 processes were shown for each gene set. Blue and red panels represent down-regulated and up-regulated gene sets respectively.

Overall, the number of significantly regulated genes increased over time. For all three time points post infection, a total of 4365 genes was differentially regulated. There are more up-regulated genes than down-regulated genes at each time point (Figure 1a). The up-regulated genes (399, 1143, and 2600 genes at 6, 12, and 24 hpi respectively) are dominated by pathways and processes of the innate immune response to virus infection, followed by responses to small molecules, antigen processing and presentation, regulation of T and B cell activation, metabolism, and apoptosis (Figure 2, Table 1, and Table S1). However, most of the down-regulated genes (17, 375, and 1538 genes at 6, 12, and 24 hpi respectively) are involved in mitosis and cell proliferation (Figure 2, Table 2, and Table S1). Note none of down-regulated genes were identified in 6 hpi (Figure 2).

We then investigated the expression dynamics of the 4365 human genes over the study period (Figure 1b). Most up-regulated genes at earlier time points continued to be up-regulated at later time points. For example, 373 of the 399 up-regulated genes at 6 hpi were also up-regulated at 12 and 24 hpi. 1029 of the 1143 up-regulated genes at 12 hpi were also up-regulated at 24 hpi. Moreover, the expression levels of most up-regulated genes at later time points were comparable to or higher than earlier time points (Figure 1b), suggesting immune response to viral infection became stronger over time. Similarly, most down-regulated genes observed at earlier time points continued to be down-regulated, and the expression levels of these down-regulated genes at later time points were comparable to or lower than earlier time points. The comparison of the enriched KEGG pathways in the up-regulated gene sets showed that 36 pathways were persistently activated in the study period (Table 1), most of which are related to innate immune response to virus infection. We also observed that genes involved in cell cycle continued to be down-regulated from 12 hpi to 24 hpi (Table 2).

### 3.3. Sensing IBV Infection

**Figure 3:**
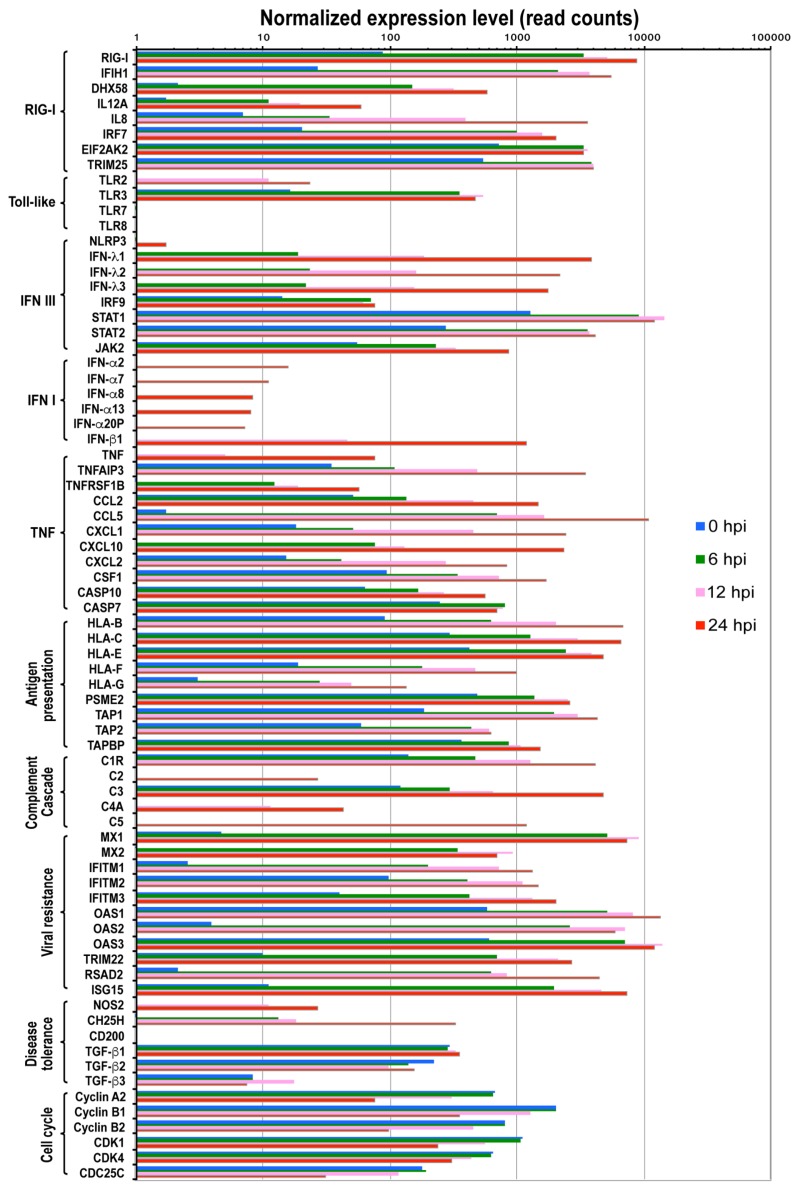
Expression levels of cellular genes in the context of functional category upon IBV infection. The functional categories and included genes with significant expression level changes upon IBV infection are shown below the bar graph. Each bar represents the average reads of 2 repeats for the gene at a time point. The orange, green, blue, and red bars indicate reads of each gene at 0, 6, 12, and 24 hpi, respectively. Differential expression statistical analysis was conducted using DESeq.

**Figure 4:**
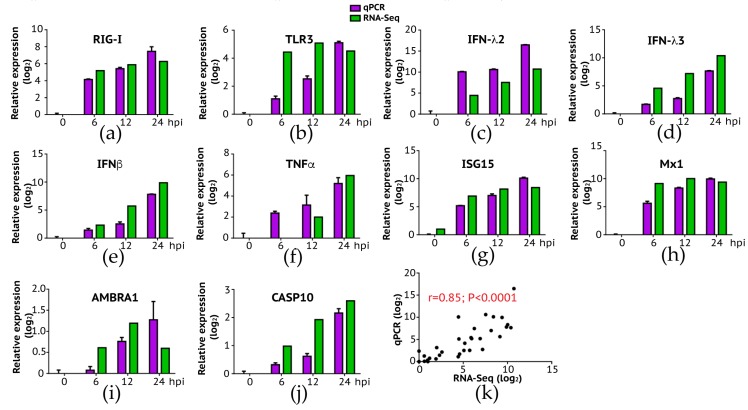
Expression levels of host genes in A549 cells verified using RT-qPCR. To validate the expression results observed in NGS, we selected a group of differentially expressed genes and performed qPCR. Overall, consistent with RNA-Seq analysis, the expression fold change of genes in innate immune response (**a**–**f**): RIG-I, TLR3, IFN-λ2, IFN-λ3, IFNβ, and TNFα; viral resistance (**g**,**h**): ISG15 and Mx1; autophagy (**i**): AMBRA1; and apoptosis (**j**): CASP10 were highly expressed in later time points. (**k**) We measured the correlation of expression level change (log_2_) between RNA-Seq and qPCR, and the result showed a high and significant correlation (Pearson’s *r* = 0.85, *p* < 0.0001).

To better understand the innate immune response to IBV infection, we analyzed regulated genes in pathways related to viral infection including detection of infection, interferon and cytokine cascade, viral myocarditis, antigen processing and presentation, and disease tolerance (Figure 3). We then verified the expression change observed in NGS using conventional RT-qPCR (Figure 4).

Previous studies demonstrated that influenza A virus infection is mainly detected by three classes of pattern recognition receptors: Toll-like receptors TLR3 (double-stranded RNA, dsRNA), TLR7 and TLR8 (single-stranded RNA, ssRNA), retinoic acid-inducible gene I (RIG-I) (5’-triphosphate RNA) and NOD-like receptor NOD-LRR- and pyrin domain-containing 3 (NLRP3). These receptors activate different downstream pathways for antiviral activities as described below. As early as 6 hpi following IBV infection, genes in the RIG-I like receptor signaling pathway and TLR3 signaling pathway were up-regulated, including DDX58 (or RIG-I), IFIH1 (or MAD5) and DHX58 (or LGP2), IL12A, IL8, IRF7, ISG15, TRIM25, and TLR3 (Figure 3).

To further validate the gene expression results analyzed by next-generation sequencing, we conducted a conventional RT-qPCR experiment where we measured quantitative changes of a subset of genes in IBV-infected A549 cells as a function of time (post-infection). Selected genes are respectively involved in the innate immune pathway (RIG-I, TLR3, IFN-λ2, IFN-λ3, IFNβ, TNF, ISG15, and Mx1), autophagy (AMBRA1), and apoptosis (CASP10). As summarized in Figure 4, our RT-qPCR experiments confirmed the up-regulation of these genes over time (Figure 4a–j), with a significant correlation between RNA-Seq and RT-PCR results (Figure 4k: Pearson correlation *r* = 0.85; *p* < 0.0001). Interestingly, we did not observe expression of TLR7 and TLR8 at all time points in A549 cells probably because both genes are not expressed in this cell line as demonstrated in a previous study [33]. Our data suggest that double-strand RNA sensing pathway including RIG-I and TLR3 is rapidly activated following IBV infection, indicating that this pathway is important in induction of the innate immune response to IBV infection in A549 cells. Future study is needed to address a role of single-strand RNA sensing TLR7 and TLR8 in anti-IBV innate immune response.

### 3.4. Interferon Response

RIG-I and TLR7 have been reported to activate type I IFN pathway. Consistent with this, we observed that genes of the JAK-STAT signaling cascade were up regulated including IRF9, STAT1, STAT2, and JAK2, suggesting that the IFN signaling pathway was activated as early as 6 hpi. It is also surprising to observe that although TLR7 was not expressed, downstream genes of TLR7 pathway were up-regulated (MyD88, IRAK, and IRF7) (Figure 3 and Table S1), which indicate there are alternative mechanisms to initiate a similar immune response at the absence of TLR7 molecule in A549 cells. Further analysis showed that both IFN-III and IFN-I pathways were activated as a function of time following IBV infection (Figure 3 and Figure 4). For example, IFN-III family genes such as IFN-λ1, IFN-λ2, and IFN-λ3 were constantly expressed over time. IFN-I family genes including IFN-β1, IFN-α20P, IFN-α8, IFN-α2, IFN-α7, IFN-α13) genes, despite relatively low level expressions at earlier time points, were significantly activated at later time points such as 12 hpi and 24 hpi (Figure 3 and Figure 4). Moreover, we did not observe expression of IFN-γ genes of the IFN II pathway. However, surprisingly, the downstream genes of the IFN II signaling pathways were still up-regulated (Figure 3 and Figure 4), suggesting other genes other than IFN-γ may also initiate IFN II signaling.

**Figure 5:**
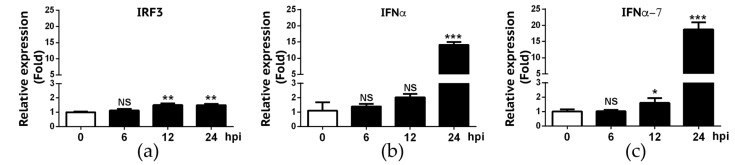
Validation of expression levels of IRF3, IFN-α and IFNα-7 in A549 cells infected by IBV. RNAs from IBV-infected A549 cells were analyzed for IRF3 (**a**), IFNα (**b**) and IFNα-7 (**c**) mRNA transcripts using RT-qPCR. Fold changes in transcript levels at the indicated time-point were calculated using the 2-ΔΔCt method so that data were normalized to both the uninfected control and the housekeeping gene GAPDH. Values represent results from three independent experiments. Statistical analysis was performed by Student’s *t* test. NS, no significant difference; *, *P* < 0.05; **, *P* < 0.01; ***, *P* < 0.001.

We also deployed the RT-qPCR approach to validate RNA-Seq data on IFN-α and IFN-α7 expression. We also included IRF-3 gene in our validation because RNA-Seq experiment did not reveal a significant change of IRF-3 gene expression in A549 cells following IBV infection. As summarized in Figure 5, RT-qPCR data showed that only approximately 1.5-fold increase of IRF-3 gene expression was found in infected A549 cells at 12 and 24 hpi, respectively. Despite statistically significant differences of IRF3 gene expression observed at 12 and 24 hpi when compared to 0h hpi (*p* < 0.01), we viewed that IRF-3 gene expression was slightly increased in A549 cells in response to IBV infection. RT-qPCR data appeared to be in agreement with the RNA-Seq result where we found that the fold-change for IRF-3 when compared to 0h hpi was 1.16 (6 hpi), 1.38 (12 hpi), and 1.14 (24 hpi), respectively (data not shown). For IFN-α and IFN-α7, similar to what was observed in RNA-Seq experiment (Figure 3), these two genes were not significantly up regulated until 24 hpi (Figure 5). Overall, these RT-qPCR data as summarized in Figure 4 and Figure 5 are in agreement with those obtained in our RNA-Seq experiment (Figure 3).

### 3.5. Cytokine and Chemokine Cascades

TNF signaling pathway, which activates NF-κB signaling pathway to induce transcription of cytokine genes and initiate apoptosis [34], was also activated in IBV-infected cells. Important players of the TNF signaling pathway, such as TNF, TNFAIP3, and TNFRSF1B, were significantly up regulated (Figure 3). Consistent with the activation of TNF signaling pathway, increased expression levels over time were observed in cytokine and chemokine genes (CCL2, CCL5, CXCL1, CXCL10, CXCL2, CSF1 etc.), autophagy genes AMBRA1 (Figure 4i), and apoptosis genes CASP10 (Figure 3 and Figure 4j, Table S1).

### 3.6. Complement Cascade

The complement system, which binds foreign antigens, forms complement-coated pathogens and destroys membrane of pathogen cells. It plays an important role in clearing foreign antigens. Our analysis revealed that many genes of the complement pathway (C1R, C2, C3, C4A, C5 etc.) were up regulated at various time points examined during IBV replication (Figure 3, Table S1).

### 3.7. Antigen Processing and Presentation

Antigen processing and presentation pathway is important for initiation of adaptive cellular and humor immune response. It has been shown that RIG-I and TLR3 can activate MHC-I system, which recruits CD8+ T cell for killing infected cells [35]. Consistent with this, starting from 6 hpi, genes of MHC-I antigen processing pathway were up-regulated about 5–20 fold including HLA-B, HLA-C, HLA-E, HLA-F, HLA-G, PSME2, TAP1, TAP2, and TAPBP (Figure 3). In contrast, TLR7/8 are reported to initiate the MHC-II antigen presentation system [36], which is required for antibody response. However, we did not observe expression of TLR7/8 in A549 cells, and consistently, not many genes in the MHC-II signaling pathway were up regulated (Table S1). Future study that addresses MHC-II response to IBV infection should be pursued in cell lines expressing TLR7/8 molecule.

### 3.8. Host Resistance and Tolerance to IBV Infection

Host resistance to viral infection or protective host response and disease tolerance play critical roles to modulate viral pathogenicity and severity of damage to host tissues and organs. Previous studies on IAV infection revealed that both processes are modulated by certain key factors [36]. Host resistance genes improve protection via repressing viral replication with multiple mechanisms, while other genes can modulate disease tolerance by depressing the inflammatory reactions. To understand whether similar mechanisms are used for host resistance and tolerance to IBV infection, we examined the expression levels of reported key regulators in protecting cells from viral infection [36]. Our analysis showed that many genes that are important for repressing viral replication and assembly were up regulated more than 100-fold (Figure 3). Furthermore, the expression levels of some well-characterized antiviral genes were significantly increased to protect cells from IBV infection. For instance, the myxovirus genes (Mx1 and Mx2), which are GTPases that block influenza A virus replication [37,38], were up regulated ~1200-fold at all three time points (Table S1). NOS2, which can induce the generation of reactive nitrogen species [39], was up-regulated about 25- and 64-fold at 12 and 24 hpi, respectively (Table S1).

### 3.9. Metabolic Processes Were Up Regulated in IBV-infected Cells at 12 and 24 hpi

The up-regulated metabolic processes are mainly involved in synthesis and metabolism of biomolecules (RNA, lipids, protein, glycan, and nitrogen compound) and regulation of protein modifications (phosphorylation and ubiquitination) (Table S1). The genes in the secretion process were also up regulated in response to IBV infection, possibly to enhance the secretion of cytokines and other extracellular molecules such as integrin and collagen. In contrast, we observed down-regulation of genes involved in synthesizing small molecules (l-serine, amino acid, and oxyacid) and their metabolic processes. Future study is needed to experimentally address the consequence of altered metabolic changes in cell physiology or in IBV replication and pathogenesis.

### 3.10. Cell Proliferation Pathway Was Repressed at 12 and 24 hpi

Genes involved in cell proliferation were dominant in down-regulated gene sets at 12 and 24 hpi (Figure 3 and Table S1). The enriched functions of these affected genes include chromosome segregation, chromosome condensation, protein complex assembly, organelle assembly, cell cycle, cell division, DNA repair, DNA recombination, and DNA replication. In particular, the significant down-regulation of cell cycle checkpoint related genes (Cyclin A2, Cyclin B1, Cyclin B2, CDK1, CDK4, and CDC25C) at 24 hpi was observed (Table S1), suggesting that IBV infection may induce cell cycle arrest, which as a result may promote cell survival to enhance IBV replication efficiency in A549 cells.

## 4. Discussion

The innate immune system is the first line of host defense against invading pathogens. Analysis of changes in this intrinsic antiviral pathway in response to IBV infection is critical to elucidate IBV-host interaction. In this study, we examined IBV infection induced response in human lung epithelial A549 cells, a well characterized cell system that responds to infection robustly and has been extensively used to investigate innate immune response of pathogens, including but not limited to Ebola virus [40], Influenza A virus [41,42,43], Aspergillus fumigatus [44], Newcastle Disease Virus [45]. Our results generated a number of interesting observations. Upon IBV infection, we observed that both type I and III IFN responses were activated over time, which appeared to be triggered by dsRNA signaling pathways including RIG-I and TLR-3, The activation of RIG-1 and IFN response during IBV infection observed here (Figure 3 and Figure 4) is similar to what has been described in previous studies [46,47]. Furthermore, a robust type III IFN response in infected-A549 cells described in this work was also reported by previous studies in antigen presentation cells [46,47], indicating that type III IFN response may serve as one of the primary antiviral defenses against influenza infection [42].

In addition, we observed no inducible expression of IFN-γ, representative of type II IFN response, which is in line with the fact that IFN-γ is only expressed in immune cells [48]. Upon activation of IFN signaling pathway, many IFN-stimulated genes (ISG) were transcribed and up regulated in IBV-infected A549 cells. These ISGs further induced inflammatory responses and activated other cellular pathways. Expression and secretion of chemokines such as IL8 would recruit neutrophil to the infection site. Induction of Mx1 protein expression would be expected to mitigate IBV replication.

Further analysis of NGS data also suggests that IBV may somehow interfere with A549 cell proliferation, which is consistent with our in vitro proliferation data reported in our previous work [32]. Previous studies showed that IAV infection arrested A549 cells at the G0/G1 phase [49,50,51], via inhibition of S phase promoting checkpoint proteins. It is assumed that cell cycle arrest can benefit viral replication. For instance, the transcription activity of DNA polymerase II (Pol II), which is required for the transcription of viral proteins, is higher in G0/G1 phase than other phases. The translation of viral proteins requires host-derived cap-dependent translation activity, which is optimal at G0/G1 phase. However, the mechanism of virus mediated host cell cycle arrest is still unclear. One hypothesis is that NS1 of IBV, similar to its counterpart in IAV, down-regulates the expression and activity of the Ras homologue gene family member A (RhoA) kinase [52], a GTPase that is critical for G1/S phase transition. Overexpression of IAV NS1 can cause increased expression of both p27 and p21, which as a result down-regulates the expression of cyclin D [52]. IAV NS1 can also inhibit the catalytic activity of pRb, CDK4, and CDK6, which are important regulators of G1/S check point. In this study, we observed decreased expression of cyclin D2, CDK4, and CDK6, while the expression of p21 was up regulated. These observations are consistent with the above hypothesis, suggesting IBV may use the same mechanism for cell cycle arrest as that reported previously for IAV. Furthermore, the expression of genes functioning in S and M phases are down regulated, implying that IBV infection may slow down cellular processes such as DNA recombination, DNA replication, and chromosome segregation. However, further investigations are required to uncover the mechanisms of these regulations and how these regulations would help IBV to replicate in cells.

Human A549 cell-specific transcriptome analysis also allowed us confirm the result from a previous study where the authors showed that TLR7 and TLR8 are not expressed in human epithelial A549 cells [33]. Lacking TLR7/TLR8 molecules apparently prevented us from investigating a function of these viral single-stand RNA recognition receptors for detection of IBV infection. Nevertheless, our study did reveal an important role of TLR3 and RIG-I in sensing IBV genome and triggering the innate immune response in infected A549 cells. Where rapid detection of IBV infection by TLR3 or RIG-1 observed in A549 cells is applicable to other epithelial cells, especially primary human epithelial cells, remains to be studied. In addition, future study also needs to consider employing epithelial cells having a full-spectrum of innate immune activators and regulators rather than TLR7/TLR8-deficient A549 cells. It is also imperative that the antiviral and cellular responses to IBV infection are evaluated in antigen-presentation cells such as macrophages or dendritic cells.

In summary, the host transcriptomic analysis presented here sheds lights on host biological systems that are affected by IBV infection. These studies shall provide novel insights into the intricate host-pathogen interaction following IBV infection of human lung epithelial cells, which can be further explored toward the elucidation of the mechanisms by which IBV remodels the epithelial environment to promote its own replication and spread in humans.

## Figures and Tables

**Table 1:** Common KEGG pathways enriched in up-regulated gene sets of 6, 12, and 24 hpi.

KEGG ID	Pathway Description	Num. of Up-Regulated Genes	False Discovery Rate
4060	Cytokine–cytokine receptor interaction	81	2.72 × 10^−20^
4062	Chemokine signaling pathway	33	0.00116
4064	NF-kappa B signaling pathway	36	3.12 × 10^−13^
4145	Phagosome	23	0.0367
4210	Apoptosis	23	2.37 × 10^−05^
4610	Complement and coagulation cascades	23	4.29 × 10^−07^
4612	Antigen processing and presentation	20	1.98 × 10^−05^
4620	Toll-like receptor signaling pathway	37	4.58 × 10^−12^
4621	NOD-like receptor signaling pathway	23	8.86 × 10^−^^09^
4622	RIG-I-like receptor signaling pathway	29	3.00 × 10^−11^
4623	Cytosolic DNA-sensing pathway	23	5.02 × 10^−08^
4630	Jak-STAT signaling pathway	49	5.03 × 10^−13^
4650	Natural killer cell mediated cytotoxicity	28	0.0001
4668	TNF signaling pathway	47	6.41 × 10^−19^
4917	Prolactin signaling pathway	16	0.00386
4940	Type I diabetes mellitus	12	0.0017
5133	Pertussis	30	4.58 × 10^−12^
5134	Legionellosis	23	2.73 × 10^−09^
5142	Chagas disease (American trypanosomiasis)	30	4.32 × 10^−08^
5143	African trypanosomiasis	11	0.00117
5144	Malaria	12	0.00552
5145	Toxoplasmosis	29	4.72 × 10^−06^
5150	Staphylococcus aureus infection	16	0.000115
5160	Hepatitis C	36	3.12 × 10^−08^
5161	Hepatitis B	37	8.17 × 10^−08^
5162	Measles	44	5.90 × 10^−13^
5164	Influenza A	58	1.79 × 10^−17^
5166	HTLV-I infection	43	0.000802
5168	Herpes simplex infection	62	6.41 × 10^−19^
5169	Epstein–Barr virus infection	33	0.00296
5203	Viral carcinogenesis	45	2.64 × 10^−08^
5320	Autoimmune thyroid disease	13	0.0032
5323	Rheumatoid arthritis	25	2.28 × 10^−06^
5330	Allograft rejection	10	0.00542
5332	Graft-versus-host disease	11	0.00245
5416	Viral myocarditis	13	0.00893

**Table 2:** KEGG pathways significantly enriched in down-regulated gene sets of 12 and 24 hpi.

Time Point	KEGG ID	Pathway Description	Num. of Down-Regulated Genes	False Discovery Rate
12 hpi	4110	Cell cycle	11	0.00193
24 hpi	3030	DNA replication	19	3.00 × 10^−11^
24 hpi	1100	Metabolic pathways	134	2.40 × 10^−07^
24 hpi	3460	Fanconi anemia pathway	17	2.40 × 10^−06^
24 hpi	4110	Cell cycle	26	1.17 × 10^−05^
24 hpi	3430	Mismatch repair	9	0.000658
24 hpi	4114	Oocyte meiosis	20	0.00135
24 hpi	260	Glycine, serine and threonine metabolism	11	0.00181
24 hpi	3440	Homologous recombination	9	0.00183
24 hpi	4540	Gap junction	16	0.00477
24 hpi	5032	Morphine addiction	16	0.00758
24 hpi	230	Purine metabolism	24	0.00878
24 hpi	3420	Nucleotide excision repair	10	0.0213
24 hpi	4961	Endocrine and other factor-regulated calcium reabsorption	10	0.0213
24 hpi	630	Glyoxylate and dicarboxylate metabolism	7	0.0237
24 hpi	3410	Base excision repair	8	0.0279
24 hpi	280	Valine, leucine and isoleucine degradation	9	0.0444
24 hpi	1230	Biosynthesis of amino acids	12	0.0444
24 hpi	4914	Progesterone-mediated oocyte maturation	13	0.0444

## Supplementary Materials

The following are available online at https://www.mdpi.com/1999-4915/12/4/383/s1, Table S1: Differentially expressed genes at 6, 12, and 24 dpi. Table S2: Primers used in qPCR.
