# Supplementary material for: Next-Generation Sequencing Analysis of Cellular Response to Influenza B Virus Infection
Source: Viruses. 2020 Mar 31;12(4):383. doi: 10.3390/v12040383 (PMC7232189; doi:10.3390/v12040383)
Supplement: Supplementary file 1 [file viruses-12-00383-s001.zip › Table S2.docx]

| **Table S2. Sequences of primers used in qPCR** | |
| --- | --- |
| **Primer name** | **Sequence 5'-3'** |
| GAPDH-RT-FWD | GGCATGGACTGTGGTCATGAG |
| GAPDH-RT-REV | TGCACCACCAACTGCTTAGC |
| RIG-I-RT-FWD | CTGGACCCTACCTACATCCTG |
| RIG-I-RT-REV | GGCATCCAAAAAGCCACGG |
| TLR3-RT-FWD | TTGCCTTGTATCTACTTTTGGGG |
| TLR3-RT-REV | TCAACACTGTTATGTTTGTGGGT |
| IFN-β-RT-FWD | ATGACCAACAAGTGTCTCCTCC |
| IFN-β-RT-REV | GGAATCCAAGCAAGTTGTAGCTC |
| IFN-λ2-RT-FWD | TCCCAGACAGAGCTCAAAACT |
| IFN-λ2-RT-REV | GTCCCCAGTCATGTCTAGTTTCA |
| IFN-λ3-RT-FWD | TAAGAGGGCCAAAGATGCCTT |
| IFN-λ3-RT-REV | CTGGTCCAAGACATCCCCC |
| Mx1-RT-FWD | GTTTCCGAAGTGGACATCGCA |
| Mx1-RT-REV | CTGCACAGGTTGTTCTCAGC |
| ISG15-RT-FWD | CGCAGATCACCCAGAAGATCG |
| ISG15-RT-REV | TTCGTCGCATTTGTCCACCA |
| TNFα-RT-FWD | CCCCAGGGACCTCTCTCTAATC |
| TNFα-RT-REV | GGTTTGCTACAACATGGGCTACA |
| AMBRA1-RT-FWD | CTGGTAGAAGATAAAACCCGGTG |
| AMBRA1-RT-REV | AGGTAGAGCGTGGACTATCCG |
| CASP10-RT-FWD | TAGGATTGGTCCCCAACAAGA |
| CASP10-RT-REV | GAGAAACCCTTTGTCGGGTGG |
